# Supplementary figures and images for: Paralog buffering contributes to the variable essentiality of genes in cancer cell lines
Source: PLoS Genet. 2019 Oct 25;15(10):e1008466. doi: 10.1371/journal.pgen.1008466 (PMC6834290; doi:10.1371/journal.pgen.1008466)

Supplemental Figure 1

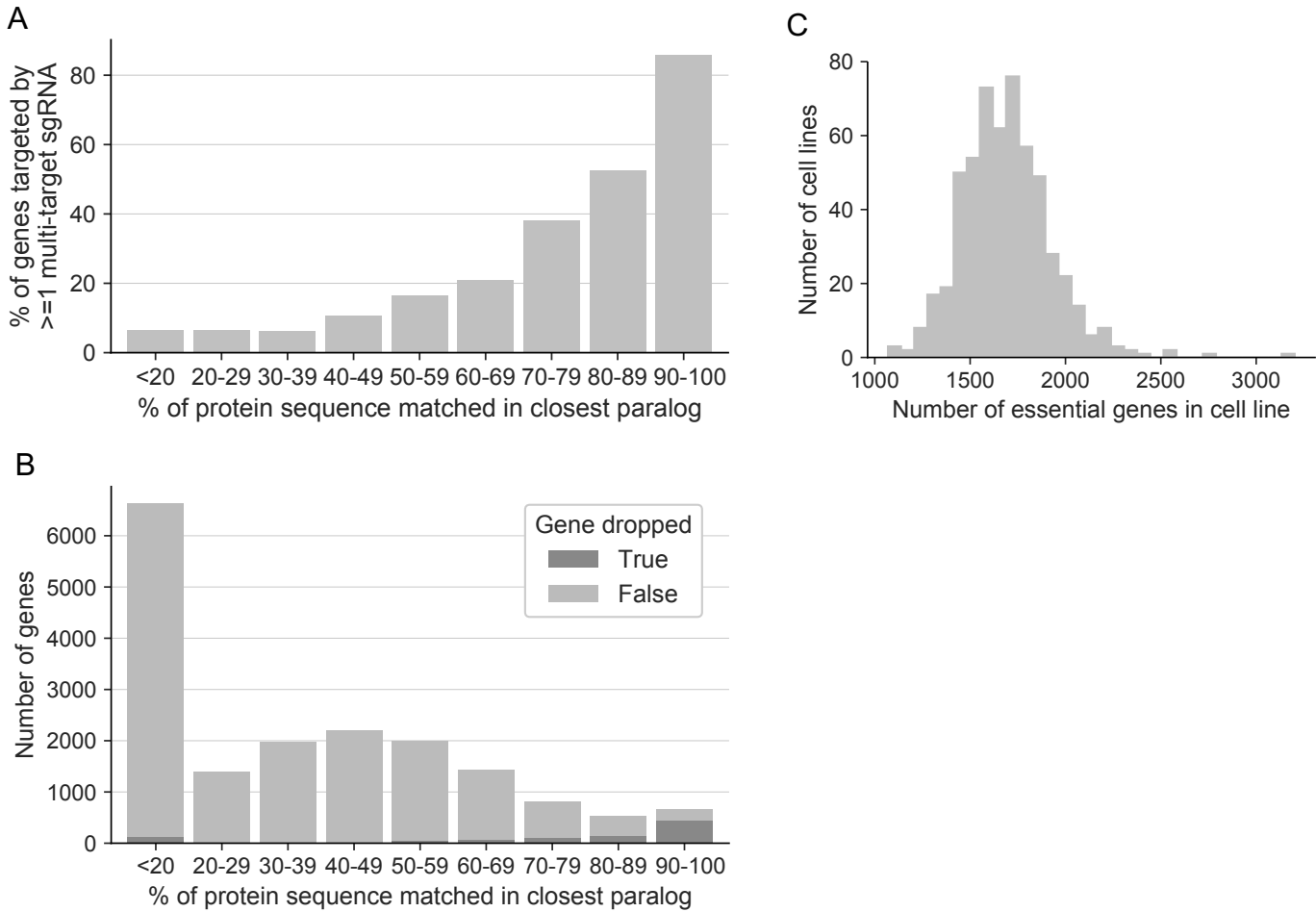

Supplement: S1 Fig — (A) Bar chart showing the percentage of genes that are targeted by at least one multi-target sgRNA for all genes targeted in the CRISPR screens binned according to the protein sequence identity they share with their closest paralog (if any). (B) Bar chart showing the number of genes that were filtered out (dropped) among all genes binned according to the protein sequence identity the genes share with their closest paralog (if any). (C) Histogram showing the distribution of the count of genes that are essential across all cell lines. (PDF) [file pgen.1008466.s006.pdf]

Supplemental Figure 2

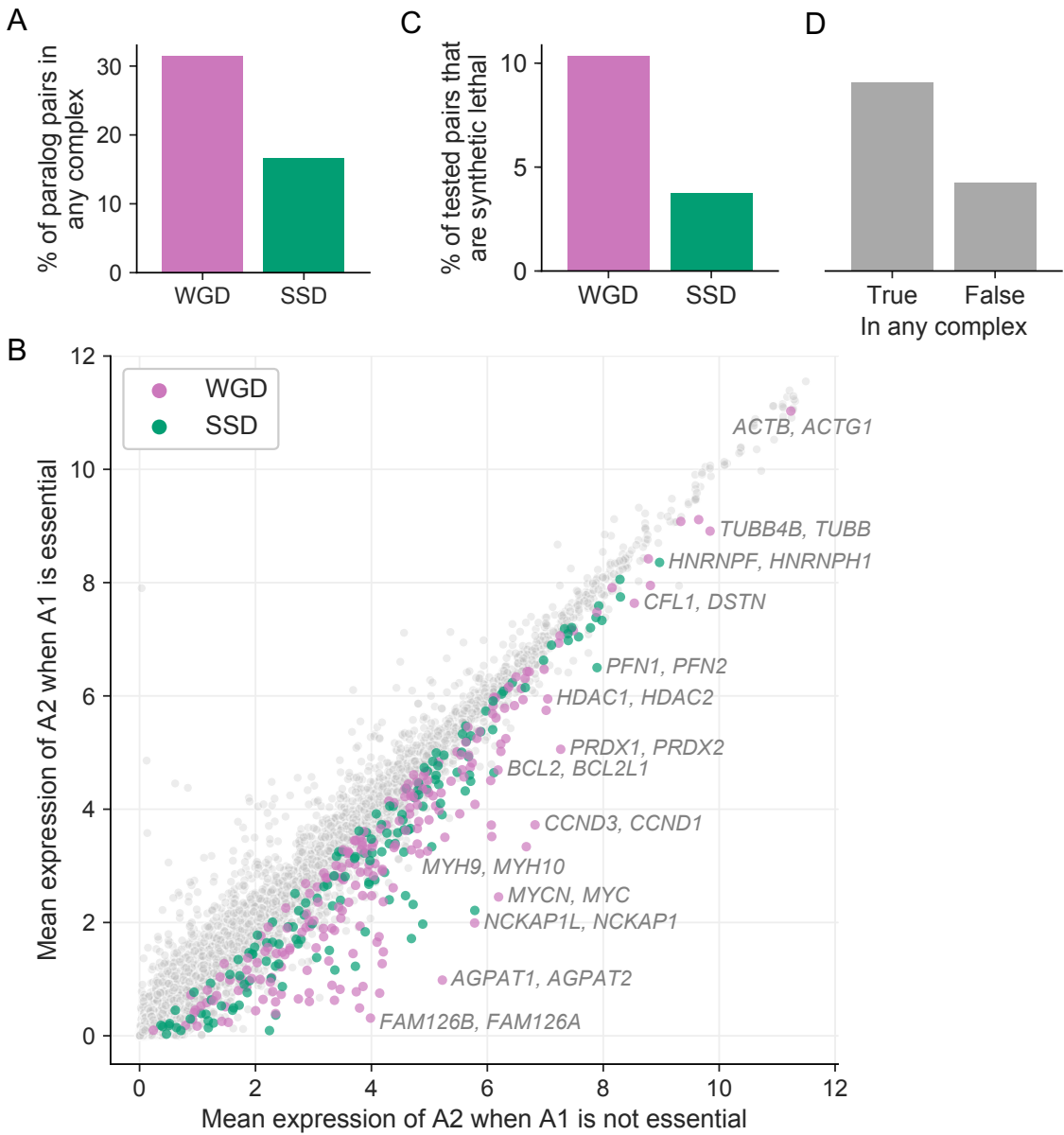

Supplement: S2 Fig — (A) Bar chart showing the percentage of WGD and SSD pairs in our dataset where at least one gene is a protein complex member. (B) Scatterplot of all tested pairs showing the mean expression of A2 in the cell lines where A1 is not essential (x-axis) or essential (y-axis). Colored dots indicate pairs for which the expression of A2 is significantly lower in the cell lines where A1 is essential, i.e. putative synthetic lethal pairs, while grey dots represent pairs that were tested but not found to be synthetic lethal. Color corresponds to duplication type: pink for WGDs and green for SSDs. Selected synthetic lethal pairs involving protein complex subunits are labelled. Similar to Fig 5B, but here all sometimes essential paralogs pairs are included, instead of just the most sequence-similar pairs. (C) Bar chart showing the percentage of WGD and SSD pairs that are synthetic lethal. Similar to Fig 6A but here all sometimes essential paralog pairs are included. (D) Bar chart showing the percentage of synthetic lethal pairs among pairs where at least one gene is a protein complex member versus among pairs where neither gene is a protein complex member. Similar to Fig 6B but here all sometimes essential paralog pairs are included. (PDF) [file pgen.1008466.s007.pdf]

Supplemental Figure 3 (threshold = -0.4)

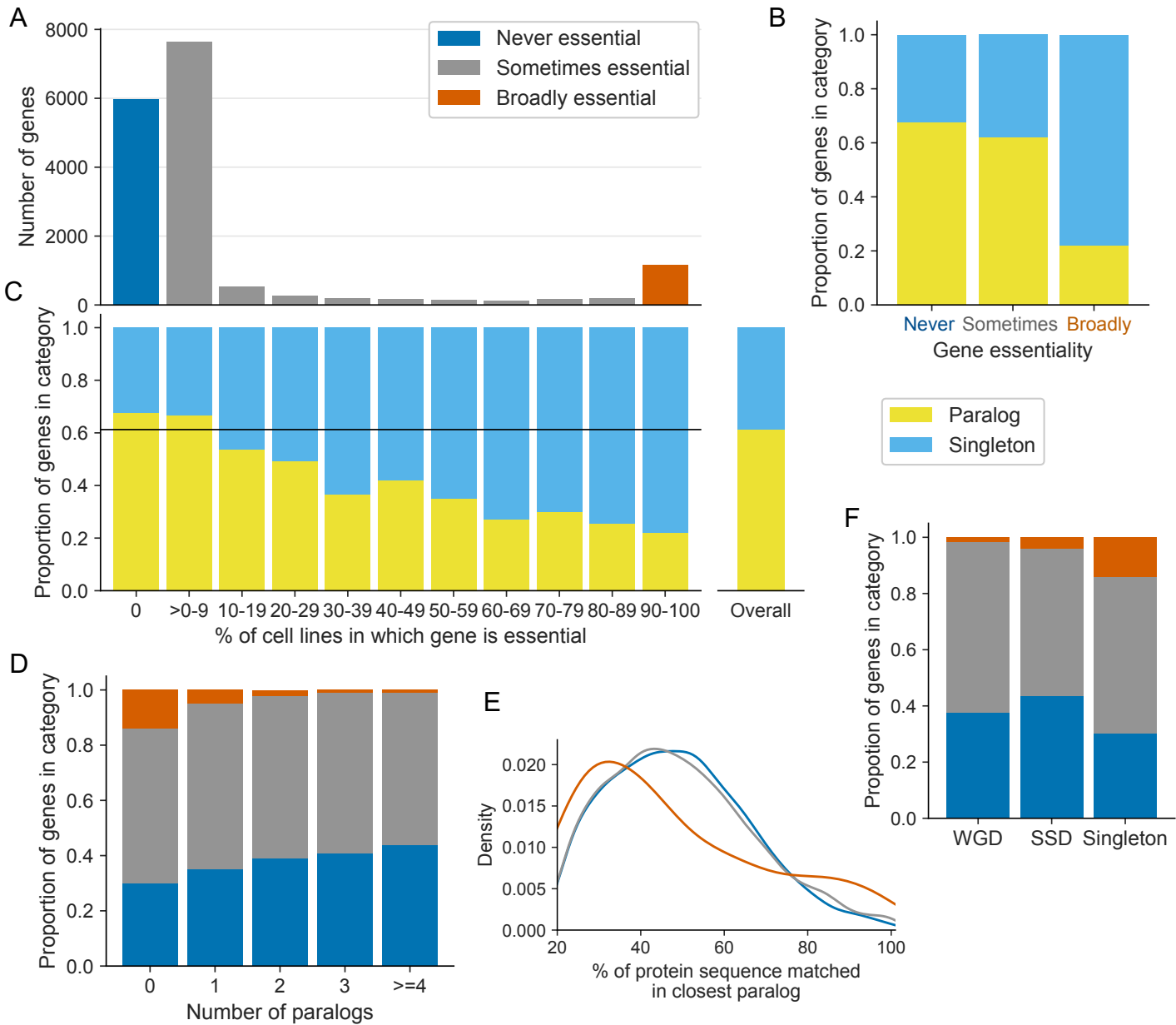

Supplement: S3 Fig — (A) Distribution of the percentage of cell lines in which a gene is essential when using a fitness score of -0.4 as the threshold for essentiality. The colors indicate the three broad categories of essentiality: genes that are essential in 0% of cell lines (blue), genes that are essential in more than 0 but at most 90% of cell lines (gray), and genes that are essential in 90% or more of the cell lines (orange). (B) Stacked bar graph showing the proportion of genes in each of the three essentiality categories that are paralogs (yellow) vs. those that are singletons (cyan). Genes that are never essential are significantly enriched in paralogs (OR = 1.5, p<2x10^-16^, Fisher’s exact test) and genes that are broadly essential are significantly enriched in singletons (OR = 6.3, p<2x10^-16^, Fisher’s exact test). (C) Stacked bar graph showing, for all genes binned according to the percentage of cell lines in which they are essential, the proportion of genes in each bin that are paralogs (yellow), or singletons (cyan). For reference, on the right is a bar showing the proportion of genes in the full dataset that are paralogs or singletons. (D) Stacked bar graph showing, for genes binned according to the number of paralogs they have, the proportion of genes in each bin that are never, sometimes and broadly essential. This proportion is significantly related to the number of paralogs (p<2x10^-16^, chi-squared test). (E) The kernel density estimates of the percent of a gene’s protein sequence that is identical in its closest paralog, for paralogs in each essentiality category. The median sequence identity for never and sometimes essential genes (~48% and ~47.6% respectively) is significantly higher than the median sequence identity for broadly essential genes (~42.1%, p = 0.002, Mann-Whitney U test). Plot is drawn using Seaborn’s kdeplot function with the default parameters. (F) Stacked bar graph showing the proportions of WGDs, SSDs and singletons that are never, sometimes and broad [file pgen.1008466.s008.pdf]

Supplemental Figure 4 (threshold = -0.6)

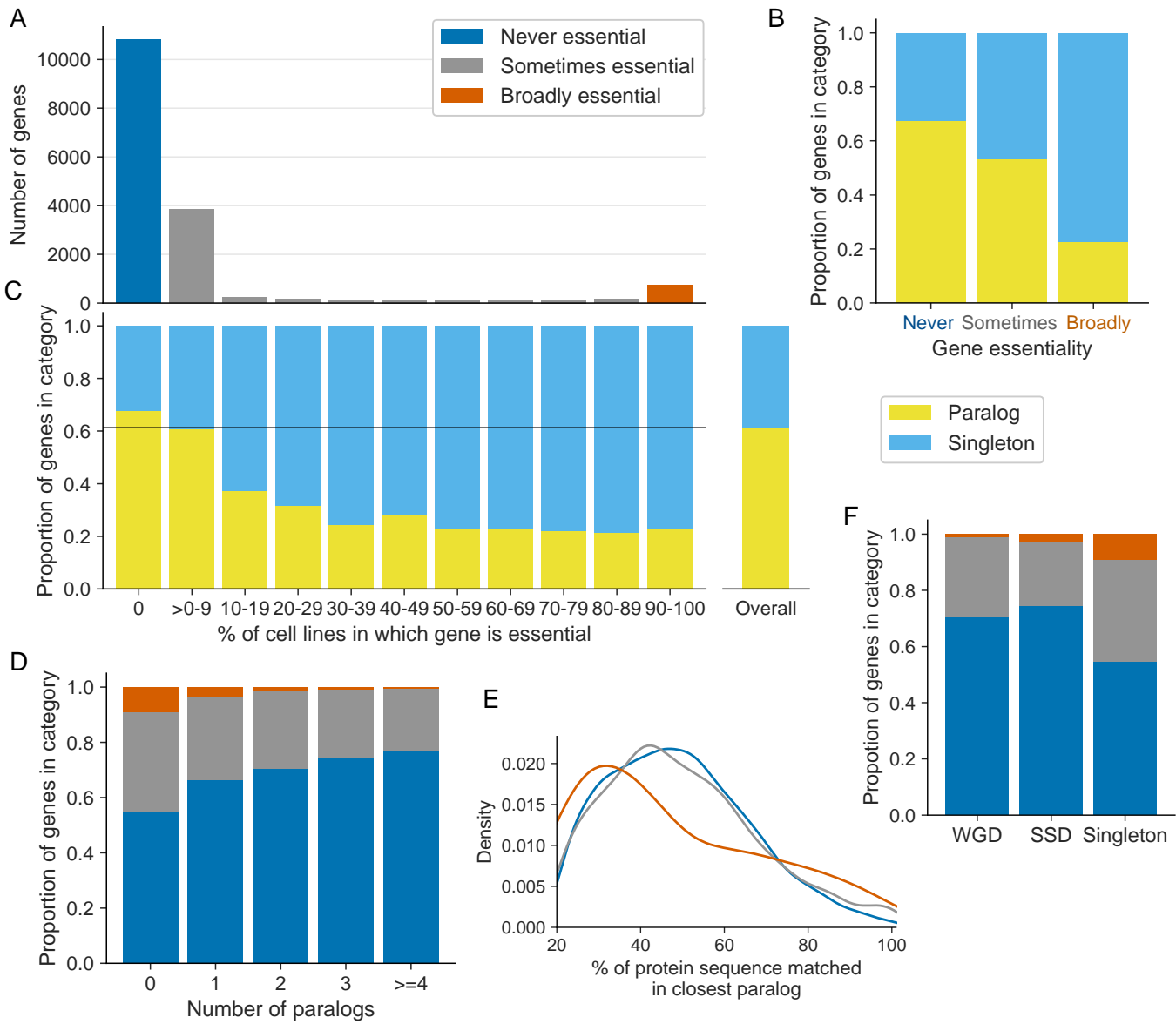

Supplement: S4 Fig — (A) Distribution of the percentage of cell lines in which a gene is essential when using a fitness score of -0.6 as the threshold for essentiality. The colors indicate the three broad categories of essentiality: genes that are essential in 0% of cell lines (blue), genes that are essential in more than 0 but at most 90% of cell lines (gray), and genes that are essential in 90% or more of the cell lines (orange). (B) Stacked bar graph showing the proportion of genes in each of the three essentiality categories that are paralogs (yellow) vs. those that are singletons (cyan). Genes that are never essential are significantly enriched in paralogs (OR = 2.1, p<2x10^-16^, Fisher’s exact test) and genes that are broadly essential are significantly enriched in singletons (OR = 5.8, p<2x10^-16^, Fisher’s exact test). (C) Stacked bar graph showing, for all genes binned according to the percentage of cell lines in which they are essential, the proportion of genes in each bin that are paralogs (yellow), or singletons (cyan). For reference, on the right is a bar showing the proportion of genes in the full dataset that are paralogs or singletons. (D) Stacked bar graph showing, for genes binned according to the number of paralogs they have, the proportion of genes in each bin that are never, sometimes and broadly essential. This proportion is significantly related to the number of paralogs (p<2x10^-16^, chi-squared test). (E) The kernel density estimates of the percent of a gene’s protein sequence that is identical in its closest paralog, for paralogs in each essentiality category. The median sequence identity for never and sometimes essential genes (~47.8% and ~47.5% respectively) is significantly higher than the median sequence identity for broadly essential genes (~41.3%, p = 0.01, Mann-Whitney U test). Plot is drawn using Seaborn’s kdeplot function with the default parameters. (F) Stacked bar graph showing the proportions of WGDs, SSDs and singletons that are never, sometimes and broa [file pgen.1008466.s009.pdf]
