# Supplementary material for: Paralog buffering contributes to the variable essentiality of genes in cancer cell lines
Source: PLoS Genet. 2019 Oct 25;15(10):e1008466. doi: 10.1371/journal.pgen.1008466 (PMC6834290; doi:10.1371/journal.pgen.1008466)
Supplement: Attachment — Submitted filename: Review PLoS Genet De Kegel 2019.docx [file pgen.1008466.s010.docx]

Review: PGENETICS-D-19-01244

The authors report a study of gene essentiality with an analysis of 100s of human cancer cell lines in which CRISPR had been used to create 1000s of mutant genes. With a reanalysis of reported data, they made several observations: (1) paralogous genes are less likely to be essential than singleton genes, (2) paralogues that are functionally more similar (based on their DNA sequence) are less likely to be essential, (3) whole-genome duplication provides more buffering than segmental duplications, (4) genes in protein complexes show greater buffering than those that are not part of such complexes, (5) lower expression of the paralogue is associated with essentiality. A key aspect of the study design is a survey of 100s of genetically heterogeneous cancer cell lines that enable tests across genetic backgrounds.

The questions are important, and the work is quite well done, and the manuscript well-written. Some of these questions have been addressed in other publications, as the authors acknowledge. The unique aspect here is the use of 100s of genetically heterogeneous cell lines.

Several issues need to be addressed:

1. Fitness needs to be defined. The authors use CERES to score fitness, but the cellular features on which the scores are based need to be described. Example – p3, line 30: ‘cause cells to die’ – is death the primary (only?) measure of fitness? How is ‘reduced fitness’ measured, or ‘moderate fitness’?

2. The use of cancer cell lines needs to be justified. As the authors acknowledge (Discussion) (p19), transformed cells undergo frequent genome rearrangements (chromosome loss, gain and translocation as well as DNA sequence changes). Each cell line not only differs in origin (DNA sequence) but also in genome organization – the nature of the heterogeneity is probably much more diverse than the difference between any two humans. These changes must also affect paralogous genes, and hence the results of any analysis, including that reported here. Could these undocumented genomic changes affect the heterogeneity in essentiality scores, e.g. conditional essential, e.g. the ‘sometimes essential’ category (page 9, line 185) if the rearrangements include paralogous genes?

Also, have the cancer cell lines been sequenced? Can these data be incorporated into the analysis so that the repertoire of paralogous genes be used for each cell line, rather than the reference genome sequence?

3. Why categorize fitness (into bins) rather than use it as a continuous measure; for example, p7, line 146 where data are categorized as severe fitness deficit, moderate and no fitness reduction), or the ten bins reported on page 10, line199). Fitness is measured on a continuous scale. Binning (categories) loses information.

Presumably, no fitness reduction means below a threshold, an undetectable fitness reduction, yes? And if yes, what’s the power to detect a fitness reduction?

4. Several questions are tested separately, for categorized data tested with t-tests etc ( throughout the Results). Examples: number of essential genes as paralogs (p10), their sequence similarity (p11), Why not use a regression model that incorporates several variables together? In this way, the contribution of variables can be assessed individually and jointly.

5. Expression analysis – a tendency to be lower in paralogues. The logic for this argument is unclear; I don’t understand why expression should be affected. Is the argument that paralogues with lower expression are more likely to be essential? Why, how? A more clear argument is needed. It’s hard a priori to understand how fitness is related to expression level.

6. An aspect that’s missing is a deeper look (discussion) about how all of this might work (mechanisms – connecting the evidence with other information about divergent and overlapping functions, cell types, etc? What do current results teach us about the more general problems? See page 18, lines 395-397, this begs for a deeper discussion about how this might work.

Other issues:

1. Several points need to be addressed:

a. Fitness in cells rather than organisms – a serious caveat to fitness and to generality.

b. In the Introduction, the authors argue that paralogues provide ‘insurance’ in case other family members acquire deleterious mutations. The common problem with this argument is that it is unclear how sequence and function for ‘insurance’ genes is maintained when ‘insured’ genes are protected from selection, - a classic problem. If ‘insurance’ is mentioned, then the way it might work should also be addressed.

c. Mention should also be made that essentiality is measured in cells in a relatively constant and defined environment. Environmental differences and flcutuations could affect essentiality.

d. WGD versus SSD – is an alternative explanation to (‘dosage balance’) that WGD provides more paralogs than a segmental duplication (or is this part of your argument that I missed)?

Grammar and writing

1. Abstract, line 2. ‘paralog genes’ should be ‘paralogous genes’.

2. Abstract, line 11. ‘sequence similar’ should be ‘sequence-similar’ – it’s a phrase hence the hyphen.

3. Abstract, line 12. ‘We determine that ….’, ‘determine’ is not the right word, ‘conclude’ maybe?

4. page 3, line 26. ‘our’? – is ‘our’ the right word?

5. Similar issues throughout…..
