# Supplementary material for: Paralog buffering contributes to the variable essentiality of genes in cancer cell lines
Source: PLoS Genet. 2019 Oct 25;15(10):e1008466. doi: 10.1371/journal.pgen.1008466 (PMC6834290; doi:10.1371/journal.pgen.1008466)
Supplement: Attachment — Submitted filename: Response_letter.docx [file pgen.1008466.s011.docx]

**UCD Systems Biology Ireland Institute**

UCD Systems Biology Ireland

University College Dublin,

Belfield, Dublin 4**,** Ireland

T +353 1 716 2411

Dear Dr. Dudley,

Many thanks for your email on the 3rd September regarding our manuscript *“Paralog buffering contributes to the variable essentiality of genes in cancer cell lines”*. We are pleased with the positive comments made by the reviewers and happy to submit a revised version of our manuscript. The majority of changes we have made to the manuscript are edits to the text to clarify issues raised by the reviewers. In addition we have added three new analyses:

1. An analysis of the robustness of our results to the threshold used to identify essential genes (resulting in two new supplementary figures, S3 and S4)
2. An analysis to assess the ability of different features of paralogs (duplication mode, sequence similarity, number of paralogs) to jointly predict essentiality class.
3. An analysis to identify how many of the synthetic lethal effects identified using gene expression profiles can be explained by copy number variation or nonsense mutations (resulting in updates to supplementary tables S4 and S5).

To guide you through the changes we have made to the manuscript we have below included a point-by-point response to each of the comments made by the reviewers. For clarity, the reviewers’ comments are in plain text and our responses in blue text.

Best,

**Dr. Colm J. Ryan**

Assistant Professor, School of Computer Science | Group Leader, Systems Biology Ireland

University College Dublin, Dublin

**Editor:**

The reviewers appreciated the work and the manuscript itself, but had several questions and suggestions that should be addressed. One major concern, which I also had after reading the manuscript, is that (as the authors know) cancer cell lines are well known to harbor extensive aneuploidy and copy number variation. How much of the background-specific non-essentiality could be explained by the presence of multiple copies of the gene or its paralog(s)?

We have now clarified in the manuscript our rationale for focusing on variation in gene expression rather than copy number as follows (page 16):

*We focused our analysis on gene expression data because many different types of genetic and epigenetic variation ultimately result in changes to mRNA abundance. This can include direct (cis) variation of a gene or its regulatory region, e.g. hypermethylation of promoter regions often results in reduced expression of associated mRNAs, copy number amplification of a gene often results in increased abundance of associated mRNA, while nonsense DNA mutations often result in reduced mRNA abundance due to nonsense-mediated decay. Variation in mRNA expression can also capture indirect trans effects, e.g. deletion of a transcription factor resulting in reduced expression of genes located on different chromosomes.*

For the case of paralogs where we do find variation in expression suggestive of a synthetic lethal relationship (i.e. A1 is essential when A2 displays lower expression) we now assess how much of this can be attributed to copy number variation as follows (page 16):

*To identify how many of the 238 putative synthetic lethal effects were driven by genetic changes to the paralog, rather than epigenetic or trans effects, we integrated both copy number profiling and exome sequencing data. We first asked how often the putative synthetic lethal effects were likely driven by copy number variation. For 87 of the 238 putative synthetic lethal pairs the copy number of A2 is also significantly lower in the cell lines where A1 is essential compared to the cell lines where A1 is not essential (p<0.05, FDR<10%, t-test). We next asked how many of the putative synthetic lethal effects could be attributed to nonsense mutations. Among the 238 putative synthetic lethal pairs, there are 92 pairs where the A2 gene is subject to a nonsense mutation in at least one of the screened cell lines. For 3 of these 92 pairs there is an enrichment of nonsense mutations in A2 in the cell lines where A1 is essential compared to the cell lines where A1 is not essential (p<0.05, FDR<10%, Fisher’s exact test). All three of these pairs (ARID1A/ARID1B, SMARCA2/SMARCA4 and STAG1/STAG2) involve tumor suppressor genes and have previously been reported in the literature [33,34,36,37].*

We reflect on these findings in the discussion as follows (page 24):

*We estimate that at least 13-17% of cases of context-dependent essentiality among paralogs can be attributed to expression variation of a paralogous gene. We detected this by exploiting the transcriptomic variation in the cell line panel. There are likely additional buffering pairs that we did not detect because the genes were under-expressed in too few of the cell lines analyzed or because reduced expression (rather than no expression) is insufficient to reveal the synthetic lethal relationship. We note that genetic variation of the coding region of the paralogous gene, via copy number variation or nonsense mutation, is only observed in a minority of the putative synthetic lethal pairs (~37%). In the majority of cases the altered mRNA expression must be due to non-coding variation, epigenetic variation or more distal trans effects and remains to be explained.*

Finally we discuss the possibility that copy number variation of a gene itself can directly impact its own essentiality in the discussion. In the interest of keeping the manuscript easier to read we have not added new analysis of these effects as we note that these ‘self-vs-self’ effects have been discussed elsewhere and apply both to singletons and paralogs. The relevant section of the discussion (page 25) reads:

*There are a number of additional explanations for the ~85% of paralogs whose variable essentiality cannot be associated with expression variation of an individual paralog. In addition to buffering relationships between paralog pairs there are likely to be higher-order buffering relationships between multiple paralogs that form part of a paralog family. Such relationships might only be revealed when three or more paralogs are perturbed simultaneously. In budding yeast these have been mapped systematically [45] but their contribution to the fitness of cancer cells remains unknown and we cannot easily detect them here. Furthermore, some of the sometimes essential paralogs may engage in synthetic lethal interactions with non-paralogous genes, i.e. genes with which they share no homology [46]. We do not have the statistical power to detect such relationships in an unbiased fashion, as it would require comparing the variable essentiality of each paralog with the expression levels of all ~20,000 protein coding genes. Finally, it is likely that some of the variable essentiality of individual genes can be attributed to genetic variation that alters the gene itself rather than its paralogs. Such self-vs-self associations include oncogene addiction effects, where mutation or amplification of a gene in a tumor cell is associated with increased dependency upon that gene for cellular fitness, and ‘CYCLOPS’ effects, where single copy (heterozygous) gene loss results in increased sensitivity of cancer cells to further perturbation of the gene itself [47].*

Other reviewer comments of particular importance are:

- the need to provide more detail about how fitness is calculated

We added more detail about how fitness is calculated as follows (page 6):

*To understand the variable essentiality of each gene we first obtained the results of genome-wide pooled CRISPR-Cas9 loss-of-function screens performed in 558 genetically heterogeneous cancer cell lines [12] (see Methods). These screens were performed using the Avana single guide RNA (sgRNA) library which contains sgRNAs targeting 17,634 genes with approximately 4 sgRNAs per gene. The fitness consequences of disrupting a specific gene are estimated by comparing the abundances of guides targeting the gene at the start of the experiment to the abundances of the same guides after 21 days of growth. A relative decrease in abundance of guides from the beginning to the end of the experiment suggests that disruption of the target gene causes a fitness defect. The magnitude of the impact on fitness can be inferred from the magnitude of change in abundance.*

- questions/ suggestions about the statistical methods used (continuous versus discretized data and t-tests versus a regression model)

We added a regression model, discussed as follows (page 14):

*Having observed that several features of paralogs influence their essentiality, we asked whether the combination of these features is more predictive of paralog essentiality than any of the features alone. To answer this question we built four logistic regression models to predict the essentiality category (never, sometimes or broadly essential) of paralogs. One model predicts using all three features (number of paralogs a gene has, the sequence identity of its closest paralog, and its mode of duplication) while the others each predict using only a single feature. We found that the full model, which incorporates all three features, is a significantly better fit for the data than any of the single-feature models (p<2x10^-16^, likelihood ratio test, for the full model vs. each single-feature model). This indicates that the combination of features explains the variation in paralog essentiality better than any of the individual features alone.*

In regards to continuous versus discretized data we note that while fitness is measured on a continuous scale, within a given context genes can be defined as essential or non-essential in a binary fashion. In yeast, strains with mutations defined as essential do not grow. There are a subset of mutants that have fitness defects that can be quantified in a continuous fashion, but there is also a binary distinction between mutations that are lethal and mutations that are tolerated. We are interested only in extreme effects here and consequently have focused on binarized data. While binning does lose data, we are not interested in more moderate fitness effects, and binning makes much of the rest of our analysis more straightforward. The obvious alternative to binning, as we have done here, would require using summary statistics (e.g. the mean or median fitness score across cell lines) that mask the variation we sought to dissect in this work.

**Reviewer #1:**

In this paper, De Kegel & Ryan investigate the contribution of paralogs to variation in gene essentiality across human cell lines. The authors re-analyze the DepMap dataset of CRISPR-Cas9 KO screens in >500 cancer cell lines to explore this question. Similar to previous studies in model organisms, they find that duplicated genes are less likely to be essential than singleton genes. They extend this finding by showing that duplicated genes also show more variation in essentiality across many different genetic backgrounds, something that has not yet been feasible to investigate in model organisms. They further investigate the underlying mechanisms driving the essentiality of genes with paralogs, such as the number of paralogs a gene has and the mode of duplication by which the paralogs occurred.

The paper is well-written, well-structured, and uses clear and precise language. All relevant data is included in the supplementary tables. The authors frequently refer to previous work done in other organisms, and - to my knowledge - cite all relevant literature. They clearly describe the used methods and have performed a thorough cleaning of the DepMap dataset before analysis.

While many of the findings in the article are intuitive given previous knowledge from model organisms, these observations have not previously been described in human cells. In addition, the authors take their work a step further than previous studies by looking at variability in gene essentiality among many different genetic backgrounds. This paper is thus certainly useful to the scientific community. However, I have a few comments, mainly about the detection and definition of differential essential genes (“sometimes essential genes”), which may affect all further downstream analyses.

We thank the reviewer for the time taken to review the manuscript.

Main comments:

1. Page 6/7 - “~86% of the genes that share 90% or more sequence identity with their closest paralog are targeted by at least one multi-target sgRNA in the Avana library”. Are there still 3 or more gRNAs remaining for these genes in the dataset, or have most of the highly similar paralogs been filtered out? If many or most of the highly similar genes have been removed, can the authors comment on how this affects the results?

As the reviewer suspects, highly sequence similar paralogs have been disproportionately filtered out as a result of dropping multi-target sgRNAs. We added acknowledgement of this, and a new supplemental figure (Fig. S1B, included below), as follows (page 7):

*We then dropped sgRNAs that target more than one gene before reprocessing all of the CRISPR screens with CERES (see Methods) which corrects for the known artefacts in CRISPR screens introduced by copy number variation [12,27]. CERES integrates data from all sgRNAs targeting a given gene to calculate a single fitness score per gene. However, after dropping the multi-targeting sgRNAs from the dataset, a number of genes were targeted with fewer than 3 sgRNAs. We excluded these genes from further analysis, resulting in a dataset containing fitness scores for 16,638 genes. As might be expected given their susceptibility to multi-targeting, genes with at least one highly sequence-similar paralog were disproportionately filtered out: ~67% of the genes that share 90% sequence identity or more with their closest paralog were dropped, while overall only ~8% of paralogs were dropped (Fig. S1B). Moreover, most of the dropped genes are highly sequence similar paralogs (Fig. S1B).*

In addition, we comment on the potential impact of these dropped genes on the results in the discussion section (page 22):

*There are additional limitations to the use of extant CRISPR screens for analyzing the essentiality of genes, especially those with a highly sequence similar paralog. Most of the genes that were filtered out as a result of discarding multi-targeting sgRNAs are highly sequence-similar paralogs, primarily those with >90% sequence identity (Fig. S1B). Our findings suggest that these genes are less likely to be broadly essential (Fig. 3C) and more likely to exhibit a synthetic lethal relationship (Fig. 6E) than paralogs with lower sequence identity. Excluding these genes may have had some influence on the results presented in this paper, but they represent a minority of all paralogs targeted (~4%), and consequently this influence is likely to be minor. To accurately assess the fitness consequences of these highly-sequence similar genes would require careful redesign of sgRNA libraries to avoid multi-targeting effects.*

2. Page 7/8 – The authors describe how they use a single CRISPR score threshold (-0.47) to determine gene essentiality. This means that genes with an average score close to this cutoff would be classified as differential essential even though the actual scores may be very comparable across cell lines (for example -0.45 in some cell lines and -0.49 in others). In addition, genes with a score just below -0.47 in one cell line and above -0.47 in all others would be classified as differential essential. This could be prevented by for example excluding all scores in the -0.45 to -0.50 range. Could the authors comment and/or show data on the effect of their choice of this single threshold on the set of differential essential genes?

To test the robustness of our results to the threshold used to identify essential genes, we have re-analyzed the data using two additional thresholds: one above (-0.4) and one below (-0.6) the original threshold of -0.47. The results of these analyses have been summarized in two new supplemental figures, Fig. S3 and Fig. S4 (included below), and are discussed as follows (page 22):

*The definition of context-specific essentiality requires a reasonable estimation of where genes are and aren’t essential. In yeast, essentiality has been determined empirically based on the viability of strains with defined gene knockouts [1]. A similar resource is not available for human cells. In this work we applied a threshold to quantitative fitness scores derived from genome-wide pooled CRISPR screens in cancer cell lines in order to call each gene in each cell line either essential or non-essential. We note that genes identified as non-essential in our analysis may be essential under some other environmental conditions and this may only become evident as screens are performed in multiple conditions. The score threshold chosen was based on a data-driven analysis of the fitness score distribution across all cell lines (Fig. 1B). However, as this threshold could potentially influence the downstream results, we also analyzed the data after binarizing the fitness scores with two additional thresholds: -0.4 and -0.6. Compared to the original threshold, these thresholds result in a larger and smaller set of sometimes essential genes, respectively, but all of the general trends that we observed regarding the relationship between essentiality and gene duplication hold (Fig. S3 and Fig. S4 for thresholds -0.4 and -0.6 respectively).*

3. Page 6-8 - If a given cell line carries a mutation (compared to the reference genome) in the sequence targeted by a guide, this guide may be less efficient in this cell line. Could the authors comment on whether some of the observed differential essentiality could be caused by differences in guide efficiency due to mutations or other sequence differences between cell lines?

We have commented on this issue in the discussion (page 23), as follows:

*A further issue that could confound the estimates of cell line specific fitness effects is that cell line specific mutations could impact the efficacy of individual sgRNAs - e.g. single nucleotide variants may disrupt the match between the sgRNA protospacer and DNA sequence - or introduce additional multi-targeting effects [24]. The sgRNAs used in the screens analyzed were designed using the human reference genome and consequently may be subject to such effects in the genetically variable set of cell lines under consideration. We expect that requiring a minimum of three different sgRNAs per gene should reduce the impact of genetic variation that alters the efficacy of individual guides or introduces off-target effects due to cell-line specific mutations. However, we cannot rule out that some of the observed context-dependent essentiality was influenced by bias in the sgRNA fitness scores resulting from cell-line specific mutations.*

Minor comments:

1. Page 13/14 - The authors use expression data to identify gene pairs where the essentiality of a gene A correlates with lower expression of its paralog B. I agree with the authors that these could indicate potential synthetic lethal relationships, but other explanations are also possible. Although at several places they describe these as “putative” synthetic lethal relationships, at other moments they talk in more definitive terms about synthetic lethal pairs. In my opinion, consistent use of more careful phrasing like “putative synthetic lethal pairs” would be preferable.

We agree with the reviewer that consistent use of ‘putative synthetic lethal pairs’ is preferable, and have updated the text accordingly.

**Reviewer #2:**

The authors report a study of gene essentiality with an analysis of 100s of human cancer cell lines in which CRISPR had been used to create 1000s of mutant genes. With a reanalysis of reported data, they made several observations: (1) paralogous genes are less likely to be essential than singleton genes, (2) paralogues that are functionally more similar (based on their DNA sequence) are less likely to be essential, (3) whole-genome duplication provides more buffering than segmental duplications, (4) genes in protein complexes show greater buffering than those that are not part of such complexes, (5) lower expression of the paralogue is associated with essentiality. A key aspect of the study design is a survey of 100s of genetically heterogeneous cancer cell lines that enable tests across genetic backgrounds.

The questions are important, and the work is quite well done, and the manuscript well-written. Some of these questions have been addressed in other publications, as the authors acknowledge. The unique aspect here is the use of 100s of genetically heterogeneous cell lines.

We thank the reviewer for the careful reading of our manuscript.

Several issues need to be addressed:

1. Fitness needs to be defined. The authors use CERES to score fitness, but the cellular features on which the scores are based need to be described. Example – p3, line 30: ‘cause cells to die’ – is death the primary (only?) measure of fitness? How is ‘reduced fitness’ measured, or ‘moderate fitness’?

To address this issue we have explained how the quantified fitness impact of disrupting a gene is derived on page 6 as follows:

*To understand the variable essentiality of each gene we first obtained the results of genome-wide pooled CRISPR-Cas9 loss-of-function screens performed in 558 genetically heterogeneous cancer cell lines [12] (see Methods). These screens were performed using the Avana single guide RNA (sgRNA) library which contains sgRNAs targeting 17,634 genes with approximately 4 sgRNAs per gene. The fitness consequences of disrupting a specific gene are estimated by comparing the abundances of guides targeting the gene at the start of the experiment to the abundances of the same guides after 21 days of growth. A relative decrease in abundance of guides from the beginning to the end of the experiment suggests that disruption of the target gene causes a fitness defect. The magnitude of the impact on fitness can be inferred from the magnitude of change in abundance.*

We note that the ‘cause cells to die’ phrase is only used in the Author Summary, intended for a lay audience. We believe discussing cellular fitness would be more confusing for this purpose.

2. The use of cancer cell lines needs to be justified. As the authors acknowledge (Discussion) (p19), transformed cells undergo frequent genome rearrangements (chromosome loss, gain and translocation as well as DNA sequence changes). Each cell line not only differs in origin (DNA sequence) but also in genome organization – the nature of the heterogeneity is probably much more diverse than the difference between any two humans. These changes must also affect paralogous genes, and hence the results of any analysis, including that reported here. Could these undocumented genomic changes affect the heterogeneity in essentiality scores, e.g. conditional essential, e.g. the ‘sometimes essential’ category (page 9, line 185) if the rearrangements include paralogous genes?

Also, have the cancer cell lines been sequenced?  Can these data be incorporated into the analysis so that the repertoire of paralogous genes be used for each cell line, rather than the reference genome sequence?

We note that the focus of this manuscript is explicitly on cancer cell lines and do not claim in the text that these are a proxy for organismal fitness. We have adjusted the text to make our rationale for focusing on gene expression rather than mutations more clear. The relevant section is as follows (page 16):

*We focused our analysis on gene expression data because many different types of genetic and epigenetic variation ultimately result in changes to mRNA abundance. This can include direct (cis) variation of a gene or its regulatory region, e.g. hypermethylation of promoter regions often results in reduced expression of associated mRNAs, copy number amplification of a gene often results in increased abundance of associated mRNA, while nonsense DNA mutations often result in reduced mRNA abundance due to nonsense-mediated decay. Variation in mRNA expression can also capture indirect trans effects, e.g. deletion of a transcription factor resulting in reduced expression of genes located on different chromosomes.*

We now also explicitly address the extent to which the synthetic lethal effects can be explained by copy number variation and nonsense mutation. We do this for both the putative synthetic lethals among the closest paralog pairs (page 16):

*To identify how many of the 238 putative synthetic lethal effects were driven by genetic changes to the paralog, rather than epigenetic or trans effects, we integrated both copy number profiling and exome sequencing data. We first asked how often the putative synthetic lethal effects were likely driven by copy number variation. For 87 of the 238 putative synthetic lethal pairs the copy number of A2 is also significantly lower in the cell lines where A1 is essential compared to the cell lines where A1 is not essential (p<0.05, FDR<10%, t-test). We next asked how many of the putative synthetic lethal effects could be attributed to nonsense mutations. Among the 238 putative synthetic lethal pairs, there are 92 pairs where the A2 gene is subject to a nonsense mutation in at least one of the screened cell lines. For 3 of these 92 pairs there is an enrichment of nonsense mutations in A2 in the cell lines where A1 is essential compared to the cell lines where A1 is not essential (p<0.05, FDR<10%, Fisher’s exact test). All three of these pairs (ARID1A/ARID1B, SMARCA2/SMARCA4 and STAG1/STAG2) involve tumor suppressor genes and have previously been reported in the literature [33,34,36,37].*

And the putative synthetic lethal among all paralog pairs (page 21):

*We again asked how often this putative synthetic lethal effect was likely driven by copy number variation or genetic mutation. For 115 of the 374 putative synthetic lethal pairs the copy number of A2 is significantly lower in the cell lines where A1 is essential compared to the cell lines where A1 is not essential (t-test, p<0.05, FDR<10%).  Analyzing nonsense mutations we found only the same three pairs as in the previous analysis (ARID1A/ARID1B, SMARCA2/SMARCA4, STAG1/STAG2) (Fisher’s exact test, p<0.05, FDR<10%).*

3. Why categorize fitness (into bins) rather than use it as a continuous measure; for example, p7, line 146 where data are categorized as severe fitness deficit, moderate and no fitness reduction), or the ten bins reported on page 10, line199). Fitness is measured on a continuous scale. Binning (categories) loses information.

Presumably, no fitness reduction means below a threshold, an undetectable fitness reduction, yes?  And if yes, what’s the power to detect a fitness reduction?

While fitness is measured on a continuous scale, within a given context genes can be defined as essential or non-essential in a binary fashion. In yeast, strains with mutations defined as essential do not grow. There are a subset of mutants that have fitness defects that can be quantified in a continuous fashion, but there is also a binary distinction between mutations that are lethal and mutations that are tolerated. We are interested only in extreme effects here and consequently have focused on binarized data. While binning does lose data, we are not interested in more moderate fitness effects, and binning makes much of the rest of our analysis more straightforward. The obvious alternative to binning, as we have done here, would require using summary statistics (e.g. the mean or median fitness score across cell lines) that mask the variation we sought to dissect in this work.

While we are explicitly interested in essentiality, the scores available from CRISPR screens are quantitative and consequently must be discretized in some fashion. For clarity on how we discretized the fitness scores we labelled the threshold line in Figure 1B (included below).

We have added a discussion on the consequences of the threshold used for discretization and the robustness of our results to this threshold (page 22):

*The definition of context-specific essentiality requires a reasonable estimation of where genes are and aren’t essential. In yeast, essentiality has been determined empirically based on the viability of strains with defined gene knockouts [1]. A similar resource is not available for human cells. In this work we applied a threshold to quantitative fitness scores derived from genome-wide pooled CRISPR screens in cancer cell lines in order to call each gene in each cell line either essential or non-essential. We note that genes identified as non-essential in our analysis may be essential under some other environmental conditions and this may only become evident as screens are performed in multiple conditions. The score threshold chosen was based on a data-driven analysis of the fitness score distribution across all cell lines (Fig. 1B). However, as this threshold could potentially influence the downstream results, we also analyzed the data after binarizing the fitness scores with two additional thresholds: -0.4 and -0.6. Compared to the original threshold, these thresholds result in a larger and smaller set of sometimes essential genes, respectively, but all of the general trends that we observed regarding the relationship between essentiality and gene duplication hold (Fig. S3 and Fig. S4 for thresholds -0.4 and -0.6 respectively).*

This new paragraph in the discussion references two new supplementary figures, S3 and S4, that are included below:

4. Several questions are tested separately, for categorized data tested with t-tests etc ( throughout the Results). Examples: number of essential genes as paralogs (p10), their sequence similarity (p11), Why not use a regression model that incorporates several variables together? In this way, the contribution of variables can be assessed individually and jointly.

We have now built such a regression model and describe it as follows in the text (page 14):

*Having observed that several features of paralogs influence their essentiality, we asked whether the combination of these features is more predictive of paralog essentiality than any of the features alone. To answer this question we built four logistic regression models to predict the essentiality category (never, sometimes or broadly essential) of paralogs. One model predicts using all three features (number of paralogs a gene has, the sequence identity of its closest paralog, and its mode of duplication) while the others each predict using only a single feature. We found that the full model, which incorporates all three features, is a significantly better fit for the data than any of the single-feature models (p<2x10^-16^, likelihood ratio test, for the full model vs. each single-feature model). This indicates that the combination of features explains the variation in paralog essentiality better than any of the individual features alone.*

5. Expression analysis – a tendency to be lower in paralogues. The logic for this argument is unclear; I don’t understand why expression should be affected. Is the argument that paralogues with lower expression are more likely to be essential? Why, how? A more clear argument is needed. It’s hard a priori to understand how fitness is related to expression level.

To clarify, the expression values for each gene are measured from the cell lines prior to the CRISPR screens performed in those same cell lines. We are not looking at the effect of a perturbation on expression but rather whether the baseline expression of a paralog of a gene A1 (denoted A2), has some predictive power for the consequences of the perturbation (via CRISPR-Cas9) of A1.

6. An aspect that’s missing is a deeper look (discussion) about how all of this might work (mechanisms – connecting the evidence with other information about divergent and overlapping functions, cell types, etc? What do current results teach us about the more general problems? See page 18, lines 395-397, this begs for a deeper discussion about how this might work.

We note that we provide an explanation for the increased context specific essentiality of WGDs in the next paragraph of the discussion. We had incorrectly separated these paragraphs in the original manuscript and have now merged them into one paragraph that reads as follows (page 24):

*Analysis of the yeast knockout collection in a single genetic background previously suggested that WGDs were less likely to be essential than SSDs* [*[11,12]*](https://paperpile.com/c/EkhgmZ/9E3tb+7mnDP)*. Here we expand this view to show that WGDs are less likely to be broadly essential than SSDs. However we also found that WGDs are less likely to be never essential than SSDs. This suggests that WGDs are more likely to be essential in a context specific fashion than SSDs, an observation that can only be made using data from multiple genetic backgrounds. This increased context specific essentiality of WGDs can be partially explained by the increased tendency of WGDs to display synthetic lethality - many WGDs appear to only be essential when the expression of their paralog is reduced. As cancer cell lines exhibit extensive genetic and epigenetic variability, the resulting variation in expression combined with the buffering relationships between paralogs appears to contribute to the variable essentiality of WGDs.*

We refrain from discussing mechanisms of paralog buffering as we focused our analysis on whether the baseline expression of a paralog of a gene A1 can predict the fitness consequences of perturbing A1. Even in budding yeast the mechanisms of paralog buffering are not well understood^1^.

^1^ [Diss G, Ascencio D, DeLuna A, Landry CR. Molecular mechanisms of paralogous compensation and the robustness of cellular networks. J Exp Zool B Mol Dev Evol. 2014;322: 488–499.](http://paperpile.com/b/EkhgmZ/lZlP)

Other issues:

1. Several points need to be addressed:

a. Fitness in cells rather than organisms – a serious caveat to fitness and to generality.

We note that the focus of this manuscript is explicitly on cancer cell lines and do not claim in the text that these are a proxy for organismal fitness.

b. In the Introduction, the authors argue that paralogues provide ‘insurance’ in case other family members acquire deleterious mutations. The common problem with this argument is that it is unclear how sequence and function for ‘insurance’ genes is maintained when ‘insured’ genes are protected from selection, - a classic problem. If ‘insurance’ is mentioned, then the way it might work should also be addressed.

Again we feel this may be confusion with regards to the nature of the ‘Author summary’ which is intended for a lay audience. We reference ‘backup’ genes in the author summary (using the analogy of a spare tire) but do not refer to ‘insurance’ or similar in the main text.

c. Mention should also be made that essentiality is measured in cells in a relatively constant and defined environment. Environmental differences and fluctuations could affect essentiality.

We mention this as follows in the discussion (page 22):

*The definition of context-specific essentiality requires a reasonable estimation of where genes are and aren’t essential. In yeast, essentiality has been determined empirically based on the viability of strains with defined gene knockouts [1]. A similar resource is not available for human cells. In this work we applied a threshold to quantitative fitness scores derived from genome-wide pooled CRISPR screens in cancer cell lines in order to call each gene in each cell line either essential or non-essential. We note that genes identified as non-essential in our analysis may be essential under some other environmental conditions and this may only become evident as screens are performed in multiple conditions.*

d. WGD versus SSD – is an alternative explanation to (‘dosage balance’) that WGD provides more paralogs than a segmental duplication (or is this part of your argument that I missed)?

Unfortunately we are not clear on what is meant by this point. For all analyses where we claim an enrichment (e.g. WGDs are more variably essential than SSDs) we take into account the background frequency of each gene type, i.e. we claim WGDs are **more likely** to be conditionally essential than SSDs not that they make up the majority of conditionally essential paralogs in absolute terms. Similarly for the synthetic lethal pairs we state that WGD pairs are **more likely** to be synthetic lethal than SSDs, we are not simply observing that more WGDs are synthetic lethal than SSDs.

Grammar and writing

1. Abstract, line 2. ‘paralog genes’ should be ‘paralogous genes’.

We have simplified the abstract so this now simply reads ‘paralogs’.

2. Abstract, line 11. ‘sequence similar’ should be ‘sequence-similar’ – it’s a phrase hence the hyphen.

We have updated this and other occurrences of the phrase ‘sequence similar’ to ‘sequence-similar’ as suggested.

3. Abstract, line 12. ‘We determine that ….’, ‘determine’ is not the right word, ‘conclude’ maybe?

 We changed this line to read ‘...we find that…’, as the sentence is about an additional finding, rather than a conclusion (page 2).

4. page 3, line 26. ‘our’? – is ‘our’ the right word?

We have dropped the ‘our’ from the phrase ‘our evolutionary history’ (page 3).

5. Similar issues throughout…..

We have carefully checked the manuscript for any remaining grammar issues.

**Reviewer #3:**

In this study, De Kegel and Ryan examine the set of genes required for viability across 558 tumor cell lines, as determined by the DepMap project at the Broad institute. The authors investigate why certain genes are essential in all, some or none of the tested cell lines and to what extent this variability can be explained by gene duplication/paralogy. The authors report that, while paralogs are overall less likely to be essential, the ones that do have an essential phenotype in at least one context provide insight into the mechanisms of variable essentiality. Specifically, the probability of a gene being required for viability depends on the number of its cognate paralogs, their mutual sequence similarity and relative expression levels, as well as the nature of the duplication event they originated from.

I found this study to be very well designed, executed and described. The question posed is very interesting and takes great advantage of an extensive body of work in model organisms (i.e., yeast) as well as the latest technologies that have recently generated relevant datasets for human cancer cell lines. The analysis is thorough and rigorous, and the description of the results and interpretations is very clear and precise. I think this would be an important contribution to the field of functional genomics and cancer biology.

We thank the reviewer for the time taken to review the manuscript.

I don't have any major comments or suggestions on the manuscript.

A few minor comments are:

- The authors estimate that 13-17% of paralogs that are essential in some, but not all, cell lines are explained by their genetic dependence on a paralog partner that's variably expressed across the cell lines. Could the authors provide their intuition/speculation/preliminary insight into what could explain the other (more substantial) fraction of variably essential paralogs? Could it be synthetic lethality with other non-paralog genes that are also variably expressed across the cell lines? And if the genome-wide expression data for these cell lines is available, could one attempt to identify these synthetic lethal partners based on the correlation of their expression levels with fitness levels?

We discuss potential buffering pairs that we missed, which could make up some of the remaining percentage, as follows (page 25):

*There are a number of additional explanations for the ~85% of paralogs whose variable essentiality cannot be associated with expression variation of an individual paralog. In addition to buffering relationships between paralog pairs there are likely to be higher-order buffering relationships between multiple paralogs that form part of a paralog family. Such relationships might only be revealed when three or more paralogs are perturbed simultaneously. In budding yeast these have been mapped systematically [45] but their contribution to the fitness of cancer cells remains unknown and we cannot easily detect them here. Furthermore, some of the sometimes essential paralogs may engage in synthetic lethal interactions with non-paralogous genes, i.e. genes with which they share no homology [46]. We do not have the statistical power to detect such relationships in an unbiased fashion, as it would require comparing the variable essentiality of each paralog with the expression levels of all ~20,000 protein coding genes. Finally, it is likely that some of the variable essentiality of individual genes can be attributed to genetic variation that alters the gene itself rather than its paralogs. Such self-vs-self associations include oncogene addiction effects, where mutation or amplification of a gene in a tumor cell is associated with increased dependency upon that gene for cellular fitness, and ‘CYCLOPS’ effects, where single copy (heterozygous) gene loss results in increased sensitivity of cancer cells to further perturbation of the gene itself [47].*

- Maybe I missed it throughout the text and the methods, but could the authors clarify where is their synthetic lethality data coming from? E.g., lines 290-294: "we found that among WGD paralog pairs, ~16.5% are synthetic lethal" -- what dataset was used to perform this assessment?

We have rephrased that section for clarity as follows (page 17):

*We had already noted that WGDs are more variable in their essentiality than SSDs. A potential explanation for this increased variability is that WGDs are more likely to buffer each other’s loss than SSDs. Indeed, in yeast it has been shown that WGD pairs are more likely to be synthetic lethal than SSD pairs [22]. To test if this is the case across the panel of cancer cell lines, we analyzed the set of 1,819 paralog pairs, of which we identified 238 as putative synthetic lethal using transcriptomics, to see if they were enriched in paralogs derived from a specific duplication mode. We found that ~16.5% of WGD pairs in this set are putative synthetic lethal, while only ~7.9% of the SSD pairs in the set are putative synthetic lethal (Fig. 6A, OR=2.3, p<1x10^-7^, Fisher’s exact test). This suggests that WGD paralogs are significantly more likely to buffer each other’s loss than SSD pairs.*

- Also, lines 303-305: "Approximately 19.1% of the paralog pairs consisting of at least one protein complex member gene are synthetic lethal, compared to only ~9.3% of the non-protein-complex pairs" -- what is the overall frequency of synthetic lethality among complex- and non-complex pairs (regardless of paralogy)?

We have also slightly rephrased that section for clarity as follows (page 18):

*We noted that many of the putative synthetic lethal interaction pairs identified involved genes known to encode protein complex subunits, including the majority of the pairs known from the literature. For example ARID1A/ARID1B and SMARCA2/SMARCA4 both function as part of the SWI/SNF protein complex [40] while STAG1/STAG2 function as part of the cohesin complex [36]. This suggests that paralogs coding for protein complex subunits may be more likely than other genes to show evidence of a synthetic lethal relationship. To test this hypothesis systematically, we integrated the set of 1,819 paralog pairs with a set of manually curated protein complexes [41], and found that there was a significant overlap between putative synthetic lethality and protein complex membership. Approximately 19.1% of the paralog pairs that consist of at least one protein complex member gene are putative synthetic lethal, compared to only ~9.3% of the non-protein-complex pairs (Fig. 6B, OR=2.3, p<1x10^-8^, Fisher’s exact test). In addition, of the pairs where both paralogs are members of the same protein complex, ~26.2% are putative synthetic lethal, compared to ~12.1% of pairs where this is not the case (OR=2.6, p<1x10^-4^, Fisher’s exact test).*
